# Identifying Hub Genes Associated with Neoadjuvant Chemotherapy Resistance in Breast Cancer and Potential Drug Repurposing for the Development of Precision Medicine

**DOI:** 10.3390/ijms232012628

**Published:** 2022-10-20

**Authors:** Trishna Saha Detroja, Rajesh Detroja, Sumit Mukherjee, Abraham O. Samson

**Affiliations:** 1 The Azrieli Faculty of Medicine, Bar-Ilan University, Safed 1311502, Israel; 2 Princess Margaret Cancer Center, University Health Network, Toronto, ON M5G 2C4, Canada; 3 Department of Computer Science, Ben-Gurion University, Beer-Sheva 8410501, Israel

**Keywords:** breast cancer, neoadjuvant chemotherapy (NAC) resistance, co-expression, drug repurposing, precision medicine

## Abstract

Breast cancer is the second leading cause of morbidity and mortality in women worldwide. Despite advancements in the clinical application of neoadjuvant chemotherapy (NAC), drug resistance remains a major concern hindering treatment efficacy. Thus, identifying the key genes involved in driving NAC resistance and targeting them with known potential FDA-approved drugs could be applied to advance the precision medicine strategy. With this aim, we performed an integrative bioinformatics study to identify the key genes associated with NAC resistance in breast cancer and then performed the drug repurposing to identify the potential drugs which could use in combination with NAC to overcome drug resistance. In this study, we used publicly available RNA-seq datasets from the samples of breast cancer patients sensitive and resistant to chemotherapy and identified a total of 1446 differentially expressed genes in NAC-resistant breast cancer patients. Next, we performed gene co-expression network analysis to identify significantly co-expressed gene modules, followed by MCC (Multiple Correlation Clustering) clustering algorithms and identified 33 key hub genes associated with NAC resistance. mRNA–miRNA network analysis highlighted the potential impact of these hub genes in altering the regulatory network in NAC-resistance breast cancer cells. Further, several hub genes were found to be significantly involved in the poor overall survival of breast cancer patients. Finally, we identified FDA-approved drugs which could be useful for potential drug repurposing against those hub genes. Altogether, our findings provide new insight into the molecular mechanisms of NAC resistance and pave the way for drug repurposing techniques and personalized treatment to overcome NAC resistance in breast cancer.

## 1. Introduction

Most clinical practices offer chemotherapy as a treatment for cancer. However, the resistance to chemotherapy prevailed as a bottleneck in the advancement of cancer treatment, despite the development of various chemotherapeutics and targeted small molecule drugs [1]. In addition to showing remarkable improvement at the initial stage of treatment, drug resistance occurs over a short time, resulting in reduced anti-tumor efficacy. Recent studies show that tumors contain a high degree of cellular heterogeneity [2], which drives drug-resistant cancer evolution. In this scenario, drug-resistant cancers evolve through the therapy-induced selection of a minor resistant subpopulation of cancer cells from the original tumor [3]. Drug-resistant cancer cells generate cell plasticity by adapting their transcriptomic signature and interactome toward a phenotypic state which no longer depends on the drug-targeted pathway [4,5,6,7]. Hence, identifying the genes, pathways, and molecular interactions altered during drug resistance would be useful for developing precision medicine against cancer.

Breast cancer is the second-highest cancer-related cause of death in women, after lung and bronchus [8,9,10]. Due to its highly heterogeneous nature, chemotherapy resistance is a major challenge for breast cancer treatments [11]. Taxanes, such as paclitaxel and docetaxel (alkaloids derived from the bark of pacific yew), are neoadjuvant chemotherapeutic agents known to be extensively used to treat breast cancer [12]. Notably, they play multiple mechanisms of action to exert an anti-tumor effect. Both these drugs interfere with microtubule dynamics, which leads to mitotic arrest and apoptosis [13,14,15]. Paclitaxel and docetaxel also deplete mitochondrial calcium ions reserve to initiate cytochrome C-mediated apoptosis [16,17]. However, paclitaxel and docetaxel treatment often develop acquired resistance, resulting in chemotherapy failure and consequent cancer relapse and mortality in breast cancer patients [16,18,19,20,21]. Numerous studies have been conducted to elucidate the multifaceted mechanisms of paclitaxel and docetaxel-associated resistance. Gupta et al. have shown that upregulation of HER2/β-catenin signaling causes breast cancer paclitaxel resistance [22]. Other studies have shown that overexpression of glutaminase 1 (GLS1) [23] and downregulation of α-1,3-Mannosyl-Glycoprotein 4-β-N-Acetylglucosaminyltransferase A (MGAT4A) [24] are associated with paclitaxel resistance in breast cancer. Moreover, Chi et al., and Lai et al. showed that actin-binding protein CapG mediated activation of the PI3K/Akt pathway [25] and upregulation of the TAZ-TEAD-Cyr61/CTGF signaling pathway [26] also accounts for the breast cancer paclitaxel resistance, respectively. Other studies have shown that under-expression of the p27 protein and overexpression of kinesins lead to docetaxel resistance in breast cancer [27,28]. Although several studies are attempting to understand the molecular mechanisms behind the acquired resistance to neoadjuvant chemotherapy (NAC) in breast cancer, much is unknown about the molecular interactions and genes crucial for developing the resistance.

Understanding the molecular interactions behind NAC chemotherapy resistance in breast cancer is important to designing precision medicine. Particularly, identifying genes that could activate other compensatory pathways by changing their expression patterns and altering the protein interaction network could be important for the development of chemotherapy resistance. Therefore, comparative analysis of the gene expression data from patients of resistance and sensitivity to a particular treatment in cancer using bioinformatics and systems biology approaches could facilitate the identification of novel therapeutic targets to overcome drug resistance. With this aim, in the present study, we used 243 publicly available RNA-seq samples from sensitive and resistant to NAC treatment breast cancer patients to identify the hub genes that could be crucial for developing resistance. Further, single cell RNA-seq analysis, mRNA–miRNA interaction analysis, pathway enrichment analysis, overall survival analysis, and drug–gene interaction analysis were performed on these hub genes to identify novel therapeutic targets and their potential drug candidates, which might be useful to treat NAC-resistant breast cancer.

## 2. Results

### 2.1. Differential Gene Expression Analysis of Breast Cancer Patients Sensitive and Resistant to Chemotherapy

**Figure 1:**
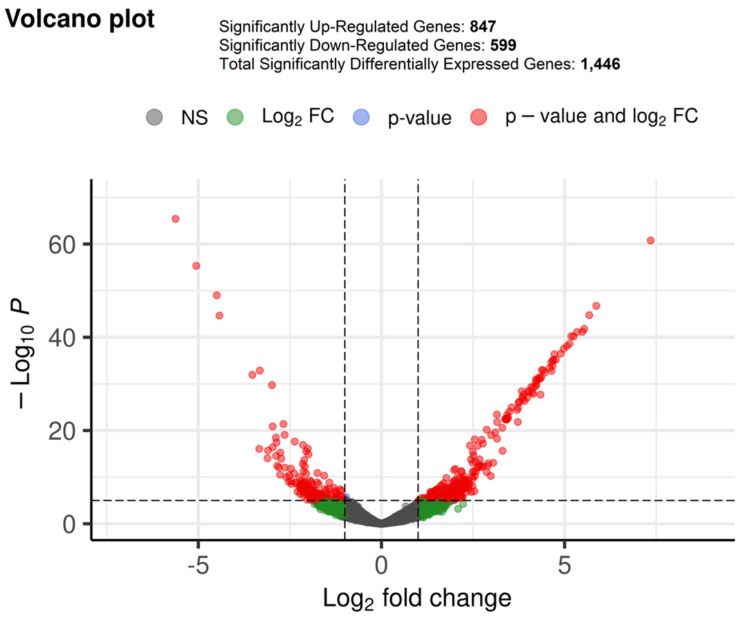
The volcano plot depicts the differentially expressed genes in breast cancer patients resistant to chemotherapy treatment.

Differential gene expression analysis was performed on between 89 and 154 samples from breast cancer patients sensitive and resistant to chemotherapy treatment, respectively. Remarkably, a total of 847 genes were found to be significantly upregulated in resistant samples (*p*-value ≤ 0.05 and log2FoldChange ≥ 1), while, a total of 599 genes were found to be significantly downregulated in resistant samples (*p*-value ≤ 0.05 and log2FoldChange ≤ −1) (Figure 1). The significantly dysregulated genes were further used for downstream analysis to identify potential hub genes crucial for chemotherapy resistance.

### 2.2. Co-Expression Network Analysis to Identify the Functionally Significant Co-Expressed Module

**Figure 2:**
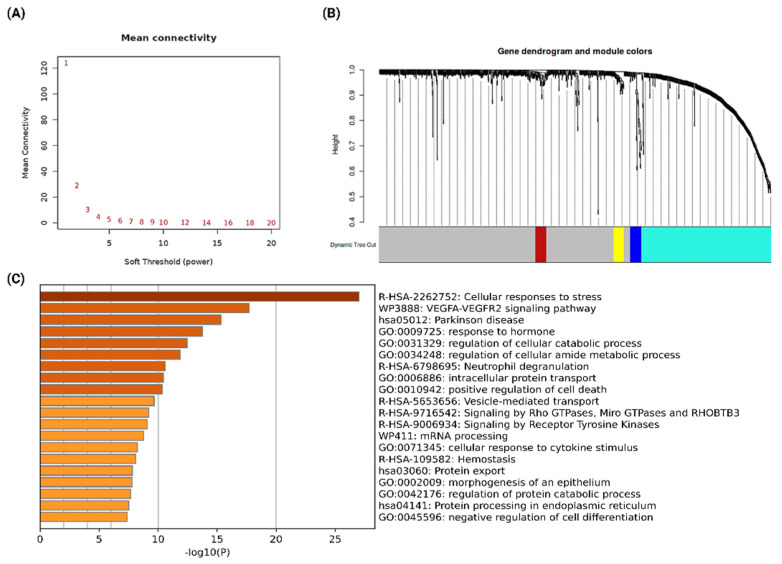
(**A**) Scale independence and mean connectivity of various soft-thresholding values (β). (**B**) Hierarchical clustering dendrograms of identified modules of co-expressed differentially expressed genes in NAC-resistant breast cancer patients. Four co-expression modules were detected and are colored blue, brown, yellow, and turquoise. (**C**) Enriched biological processes and pathways of the genes from the turquoise module. Bar graph of enriched terms across input gene lists, colored by *p*-values. The graph was prepared using Metascape.

Co-expression network analysis is important to identify the set of functionally relevant genes which have a trend to show a coordinated expression pattern across the samples [29,30,31]. In this analysis, the set of genes with similar expression patterns is assigned to a specific module. To identify the co-expressed genes from the differentially expressed genes in resistant samples, we performed a weighted gene co-expression network analysis (WGCNA). In the analysis, the soft threshold power of β was 5 when the scale-free topology model fit R2 was maximized (Figure 2A). Then, the co-expression modules were identified by the dynamic tree-cut procedure using the dynamic branch-cutting algorithm with a robust measure of interconnectedness, using DynamicTreeCut and the WGCNA R library. A total of four co-expression modules were identified in the analysis, with each module being assigned a unique color label, as represented in Figure 2B (blue, brown, yellow, and turquoise). Among 1446 differentially expressed genes, only 413 genes were assigned to different modules. This observation indicates that most of the differentially expressed genes are the results of phenotypic changes in cancer cells due to the development of drug resistance. However, those genes co-expressing and functionally involved in similar biological processes and pathways could cause drug resistance development. We identified 330 genes in the turquoise module, 29 genes in the blue module, 28 genes in the brown module, and 26 genes in the yellow module.

Further, functional enrichment analysis of significant co-expressed modules was performed using Metascape to understand the functional association of each module with different biological processes and pathways. Blue, brown, and yellow modules did not show any significant functional enrichment as the number of genes is low (<30 genes per module). We found only the turquoise module to be significantly enriched in various biological processes and pathways, which are crucial for drug resistance to cancer development (Figure 2C). The most enriched process was “cellular responses to stress”, which indicates that these genes are differentially expressed in the resistant group to generate the stress response that leads to the induction of resistance to NAC treatment. Further, we found that the most enriched signaling pathways are the VEGF-VEGFR2 signaling pathway and signaling by Receptor Tyrosine Kinases. The VEGF-VEGFR2 signaling pathway appears to mediate cellular responses involved in vasculogenesis and angiogenesis [32], whereas Receptor Tyrosine Kinases signaling is crucial for cell-to-cell communication, and controlling a broad range of complex biological functions, including cell growth, motility, differentiation, and metabolism [33]. Dysregulation of both of these signaling processes is important for cancer development, resistance, and metastasis. Therefore, alternations of these two major signaling pathways in NAC resistance to breast cancer promote drug-resistant cancer evolution. Hence, with the importance of the turquoise module, we considered genes from this module for downstream analysis.

### 2.3. Network Analysis of Co-Expressed Modules, Identification of Hub Genes from the PPI Network, and Functional Enrichment Analysis

The extracted genes from the turquoise module were used to construct the protein–protein interaction (PPI) network and hub genes identification. The genes were used as the seed to construct high confidence (0.7) PPI network on the STRING database [34]. The resultant network from the turquoise module had 321 nodes and 442 edges (PPI enrichment *p*-value < 1.0 × 10^−16^), which signifies the robustness of the turquoise module. Further, the PPI network of the turquoise module was subjected to Cytoscape v3.8.0 for visualization and hub gene identification.

**Figure 3:**
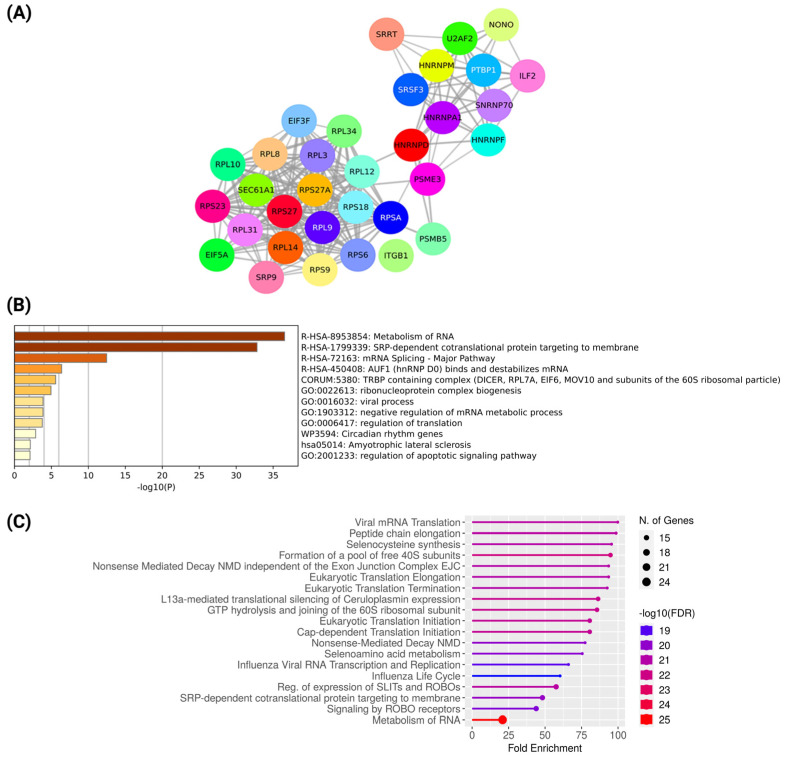
(**A**) Protein–protein interaction network of hub genes associated with NAC resistance in breast cancer. The plot was prepared using STRING. (**B**) Significantly enriched biological processes and pathways associated with the hub genes. Enrichment analysis performed using Metascape. (**C**) Significantly enriched reactome pathways associated with the hub genes. Enrichment analysis was performed using ShinyGO.

Hub genes are highly connected nodes in a network that play a critical role in regulating other genes present in the related biological processes and pathways. The CytoHubba MCC clustering algorithm was implemented to identify the top 10% hub genes of the turquoise module. We identified 33 genes as the hub in the network (nodes 33, edges 214, PPI enrichment *p*-value < 1.0 × 10^−16^), which are important for the development of NAC resistance in breast cancer (Figure 3A). Biological processes and pathway enrichment analysis of these hub genes were carried out in Metascape and ShinyGo v0.76 with an FDR cutoff of 0.05. Hub genes were found to be significantly enriched in a broad range of vital cellular processes (Figure 3B), such as RNA metabolism, mRNA splicing, mRNA transport, regulation of translation, and regulation of the apoptotic signaling pathway. This indicates that breast cancer cells with the altered phenotype(s) survive using a wide range of cellular processes in the face of drug-related stress that confer resistance to neoadjuvant therapy. Further, Reactome pathway analysis showed enrichment of hub genes in the regulation of expression of SLITs and ROBOs, signaling by ROBO receptors, cap-dependent translation initiation, selenocysteine synthesis, and metabolism of RNA (Figure 3C). Several studies have shown that the regulation of mRNA by binding or modulating the stability of the RNA can confer NAC resistance [35,36]. Moreover, ribosomal biogenesis was also found to be involved in cancer progression and resistance by regulating stress responses. Additionally, SLITs and ROBOs are involved in anti-tumor activity by regulating chemokine-mediated response in breast cancer; thus, regulation of the expression of SLITs and ROBOs might be associated with chemoresistance [37,38,39]. Additionally, other recent studies have demonstrated resistant cancer cells have increased selenocysteine synthesis, which protects cancer cells against oxidative stress [40,41,42]. Furthermore, the study by Liu et al. showed SRP-dependent co-translational protein targeting to the membrane and amplification of ribosomal protein involved in resistance [43].

### 2.4. mRNA-miRNA Network Analysis Reveals the Potentiality of the Hub Genes in the Alternation of Regulatory Network in NAC-Resistant Breast Cancer

**Figure 4:**
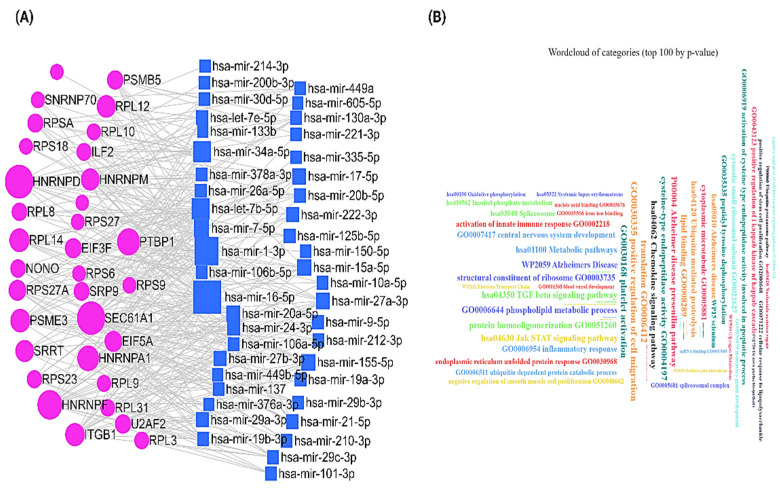
(**A**) mRNA–miRNA interaction network of hub genes. The network circle with purple color represents the hub genes, and the blue square represents the miRNA. (**B**) Top enriched processes and pathways where the miRNAs in this network are involved (*p*-adjusted value < 0.05).

To check the potentiality of these hub genes in altering the interactome of breast cancer cells, we performed mRNA–miRNA network analysis. We used miRNet 2.0 [44] to construct the mRNA–miRNA network. We found that 44 miRNAs potentially interacted with these 33 hub genes in the network (Figure 4A). All these interactions are supported by the experiments (Table S1). Next, we performed the functional enrichment of these miRNAs using miEAA 2.0 (https://www.ccb.uni-saarland.de/mieaa2, accessed on 7 October 2022) [45]. We observed that these miRNAs are significantly involved in a broad range of biological processes and pathways (*p*-adjusted value < 0.05) (Figure 4B). Therefore, alteration of the expression of the hub genes in NAC-resistant breast cancer could potentially impact the interacting miRNAs in the network, which could affect various cellular processes and pathways that could promote the changing regulatory landscape of the cancer cells.

### 2.5. Analysis of the Hub Genes in the Single-Cell RNA-seq Data of Breast Cancer Patients

**Figure 5:**
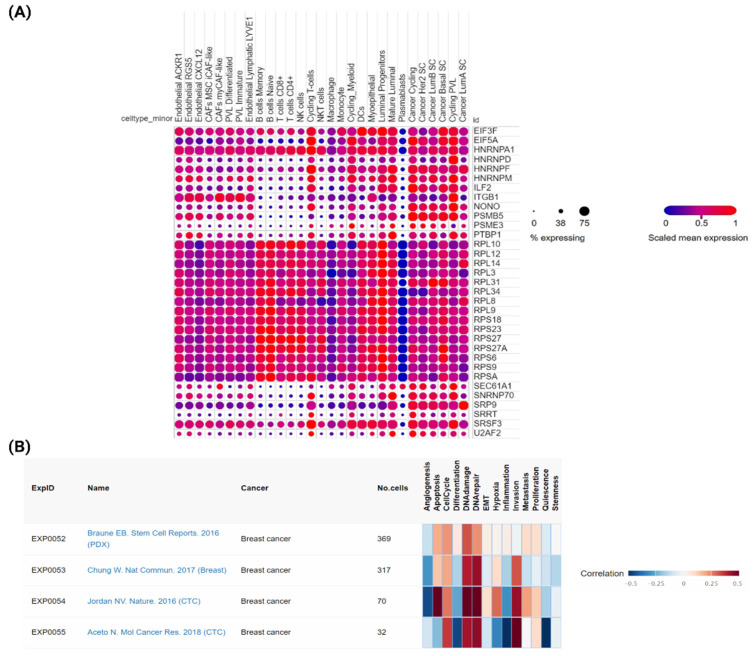
(**A**) Expression of hub genes in different cell types of scRNA-seq data of breast cancer patients from the study of Wu, S. et al. [47]. (**B**) The correlation heatmap shows the detailed information of all functional associations with hub genes in each breast cancer dataset available in CancerSEA database [46,49,50,51].

Single-cell RNA-sequencing (scRNA-seq) is a useful method to analyze disease heterogeneity at the single-cell level [46]. Understanding the pattern of cell-type-specific hub gene expression at the single-cell level could help us to determine their potentiality of being the druggable target in breast cancer. We uploaded these 33 hub genes in the study of “A single-cell and spatially resolved atlas of human breast cancers” (https://singlecell.broadinstitute.org/single_cell/study/SCP1039/a-single-cell-and-spatially-resolved-atlas-of-human-breast-cancers, accessed on 7 October 2022) [47] in the Single Cell Portal of Broad Institute to check their pattern of expression at single cell level. We observed that the pattern of expression of these hub genes is similar in most of the cell types in the tumors (Figure 5A), which suggests that targeting those hub genes could overcome the NAC resistance in breast cancer. Further, to understand the functional state of these hub genes in breast cancer cells at the single-cell level, we uploaded these 33 hub genes to the CancerSEA database (http://biocc.hrbmu.edu.cn/CancerSEA/, accessed on 7 October 2022) [48]. Interestingly, we observed that DNA damage and repair are the two most functional states in breast cancer associated with these hub genes. The gene list containing hub genes was positively correlated with these two functional states in all four scRNA-seq datasets (Figure 5B) [46,49,50,51]. DNA damage can result from chemotherapy treatment, and emerging evidence highlighted that DNA repair processes are important in drug resistance [52]. Therefore, this data suggests that the alternation of gene expression of these hub genes could promote the DNA repair process in response to NAC-mediated DNA damage and drive the development of NAC-resistant breast cancer.

### 2.6. Drug Repurposing Using Drug-Gene Interaction Analysis of Hub Genes

We found 10 hub genes out of 33 hub genes, against which 19 potential drugs (8 approved, 11 in preclinical and clinical trials) can be repurposed (Table S2). Carfilzomib and Bortezomib, used to treat hematologic malignancy, have repurposing potential against PSME3 and PSMB5 dysregulation. Ixazomib citrate, originally used in combination treatment with Lenalidomide and Dexamethasone to treat multiple myeloma, can be repurposed against PSMB5 dysregulation. Natalizumab, used in multiple sclerosis and Crohn’s disease, has the potential to be repurposed against ITGB1 dysregulation. Homoharringtonine, another drug used in hematologic malignancy, can be repurposed against RPL3 dysregulation (CMap score 100). Interestingly, antibiotics, e.g., puromycin and anisomycin, have the potential to be repurposed against RPL3 and RPL8 dysregulation. We also found drug classes such as quinoline (antimalarial), pyrimidine analogs, topoisomerase inhibitors, imatinib analog, and vinca alkaloids that can be repurposed against RPL3, RPL31, RPS9, and U2AF2 dysregulation. Mitoxantrone is used in acute nonlymphocytic leukemia and multiple sclerosis, Daunorubicin is used in acute myeloid leukemia, and Doxorubicin is used in cancer that can be used against RPSA. Moreover, the comparative toxicogenomics database revealed positive and negative associations between FDA-approved drugs and hub genes based on the literature (Table S3).

### 2.7. Survival Analysis of the Hub Genes

The Kaplan–Meier plotter web browser [53] was used for the survival analysis of 33 hub genes to check their association with survival in overall breast cancer. We identified seven hub genes (*p*-value cutoff < 0.05), of which four were upregulated hub genes (RPSA, SEC61A1, ITGB1, PSMB5) and three were downregulated hub genes (PSME3, SNRNP70, SRSF3) associated significantly with poor overall survival (Figure S1). Importantly, we found drugs with repurposing potentials such as Carfilzomib, Bortezomib, Ixazomib citrate against hub gene PSMB5, Natalizumab against hub gene ITGB1, Mitoxantrone, Daunorubicin, and Doxorubicin against hub gene RPSA, which were found to be upregulated in NAC-resistant patients, as well as associated with poor overall survival.

## 3. Discussion

Breast cancer is a broad spectrum of highly heterogeneous diseases, which pose an additional problem for developing effective treatment regimens [54]. The major obstacle faced by current treatments is drug resistance, which leads to poor patient outcomes.

NAC is the frontline treatment for treating patients with locally advanced breast cancer before tumor excision [55,56]. However, drug resistance hinders the development of efficient cancer treatment. Thus, we need a better understanding of the molecular landscape associated with NAC resistance to develop potential therapeutics. The drug repurposing approach has emerged as a promising strategy to find available drugs for designing personalized treatment, in addition to discovering new drugs [57].

In this study, we used an integrative bioinformatics approach to identify the potential hub genes that could be involved in developing NAC resistance in breast cancer. First, we downloaded the multiple publicly available RNA-seq dataset for breast cancer sensitivity and resistance to NAC. Then, we combined all the datasets, performed differential gene expression analysis, and identified 1446 differentially expressed genes (DEGs) associated with NAC resistance in breast cancer. After that, to identify the functionally relevant co-expressed genes which could be important for the development of NAC resistance breast cancer, we performed a co-expression network analysis based on the WGCNA approach. This step facilitates identifying a functionally significant co-expressed module consisting of 330 genes. Next, we performed PPI network analysis of these 330 genes and identified the top 10% of genes (33 genes) as hub genes. Then, to understand if these hub genes could alter the regulatory landscape of the cancer cells, we performed mRNA-miRNA interactome analysis. We identified 44 miRNAs that significantly interacted with these hub genes, and these miRNAs are involved in a broad range of cellular processes and pathways. Therefore, it could suggest that the alternation of gene expression of these hub genes could significantly alter the mRNA–miRNA interaction, which could impact the regulatory network of cancer cells. Then, we analyzed the functional associations of these hub genes in the scRNA-seq data of breast cancer patients, which suggest that these genes are significantly associated with the DNA repair processes in all the dataset. This finding could support that these hub genes are crucial for DNA repair processes in response to NAC-mediated DNA damage in breast cancer cells, potentially associated with the NAC resistance to breast cancer emergence. Further, we performed functional and pathway enrichment analysis, survival analysis, and drug repurposing of these hub genes.

Gene ontology and pathway enrichment analysis of 33 hub genes revealed they are involved in a broad range of cellular processes and pathways. These findings support that during the development of drug resistance to cancer emergence, cancer cells need to change their phenotype without altering their genotype, impacting the global alternations of cellular pathways and different biological processes. Therefore, these identified hub genes could be the important regulator of all the alternations of various important processes and pathways, which could lead to the development of resistance to NAC.

Next, we identified potential FDA-approved drugs against NAC-resistance associated hub where we found drugs such as carfilzomib [58], bortezomib [59], mitoxantrone [60], and homoharrington [61] used in hematologic malignancy have the potential to be repurposed against PSMB5, RPSA, and RPL3, respectively. Furthermore, the comparative toxicogenomic database provided curated FDA-approved drugs that have the positive or negative mode of action towards the differentially expressed hub genes in NAC-resistant breast cancer patients, thus can be used for developing precision medicine. Drug resistance is the key issue limiting modern cancer therapies. Drug repurposing is a promising tool that can be deployed to identify approved drugs that can be used alone or in combination with other drugs to improve treatment outcomes [62,63,64]. In this study, we have used multiple databases to screen FDA-approved drugs that have the potential to be repurposed against differentially expressed hub genes in resistance patients to overcome drug resistance.

Moreover, we performed a survival analysis of all 33 hub genes to check their impact on the survival of breast cancer patients. Here, we found four upregulated hub genes (RPSA, SEC61A1, ITGB1, PSMB5) and three downregulated hub genes (PSME3, SNRNP70, SRSF3) that are significantly associated with poor overall survival of breast cancer patients. The involvement of RPSA, SEC61A1, ITGB1, PSMB5, PSME3, and SRSF3 genes in chemotherapeutic resistance has been well-studied in various malignancies. RPSA, also known as 37LRP, was upregulated in the resistant breast cancer patient, and regarding its role in drug resistance, studies by Sun et al. have shown MGr1-Ag/37LRP confer drug resistance through FAK/PI3K and MAPK mediated pathway in gastric cancer [65]. RPSA has also been reported to evade apoptosis by impeding caspase activity in pancreatic cancer [66]. In breast and esophageal cancer, RPSA is associated with the suppression of apoptosis and autophagy [67]. RPSA was also found to promote pancreatic cancer invasion and metastasis through the MAPK signaling pathway [68]. SEC61A1 was reported to play a role in colon and hepatocellular cancer progression [69,70,71]. Hsa_circ_0007841 transcripted by SEC61A1 was found to be involved in drug resistance in doxorubicin and bortezomib-treated multiple myeloma patients [72,73]. Additionally, Cao et al. showed the involvement of the MAGI2-AS3/miR-218-5p/GDPD5/SEC61A1 axis in cisplatin resistance in nasopharyngeal carcinoma [74]. ITGB1 was identified as a predictive neo-adjuvant chemotherapy resistance marker for pathological response in breast cancer. Moreover, overexpression of ITGB1 was also shown to be associated with high matrix stiffness and poor overall survival in breast cancer patients [75]. Baltes et al. showed that ITGB1, upon binding to collagen type 1, activates the ABC efflux transporter and exerts chemoresistance in doxorubicin, cisplatin, and mitoxantrone-treated breast cancer [76]. Additionally, ITGB1 confers resistance to paclitaxel in nasopharyngeal cancer [77]. Furthermore, increased expression of ITGB1 is also involved in poor prognosis of ovarian cancer, gastric cancer, head and neck squamous cell carcinomas, and lung cancer [78,79,80,81]. Upregulated PSMB5 was found to be associated with increased cell proliferation, drug resistance, and poor prognosis in TNBC patients [82]. Similarly, Wang et al. reported the involvement of PSMB5 in enhanced cell migration and immunosuppression in breast cancer [83]. Several other studies showed an association between overexpression of PSMB5 and drug resistance in T-lymphoblastic lymphoma, myeloma, and gastric cancer [84,85,86]. PSME3 gene has lower expression in the resistance group as well as in the lower OS group. Downregulation of PSME3 is involved in cell growth arrest. Studies have shown low expression of PSME3 impedes proliferation and induces apoptosis by overexpression p53 [87,88,89]. However, Sanchez et al. found reduced p53 expression in their study despite miR-7-mediated downregulation of PSME3 expression, leading to cell cycle arrest and evasion of apoptosis [89]. Likewise, expression of anti-apoptotic proteins increases by miR-30a-3p, miR-146a-5p, and miR-491-5p dependent downregulation of PSME3, which suppresses apoptosis [3,90]. Another gene, SRSF3, was overserved with low expression in resistant patients. A recent study demonstrated a link between downregulated SRSF3 and poor overall survival [91]. SRSF3 was reported to act as a tumor suppressor in hepatocellular carcinoma and colorectal cancer. Moreover, recent studies have illustrated that lower expression of SRSF3 is associated with neoplasia progression and poor overall survival in the liver and colorectal cancer [92,93,94].

In addition, hub genes such as RPS6, RPL34, SRP9, HNRNPA1, HNRNPM, SRRT, EIF5A, NONO, and PTBP1 were significantly up or downregulated in resistant breast cancer patients but not significant as prognostic biomarkers, also reported being involved in cancer progression. Upregulation of RPS6 is involved in cancer cell proliferation, distant metastasis, and poor prognosis in breast and cervical squamous cell carcinoma [95,96,97]. Increased expression of RPL34 has been shown to be involved in malignancy progression in gastric cancer, osteosarcoma, non-small cell lung cancer, esophageal cancer, and glioma [98,99,100,101]. Rho et al. and Erdogan et al. demonstrated that the overexpression of SRP9 is involved in colorectal cancer progression and advanced breast cancer [102,103]. Increased expression of HNRNPA1 is also reported to be involved in drug resistance in pancreatic and lung cancer [104,105]. Several studies have shown that the overexpression of HNRNPM is involved in metastatic progression and increased epithelial-to-mesenchymal transition and chemoresistance [106,107,108]. Upregulated SRRT gene, also known as ARS2, is associated with increased cell proliferation and poor overall survival by modulating the miR-6734-3p/p27 axis and miR-6798-3p in myeloid leukemia and glioblastoma, respectively [109,110]. Downregulation of EIF5A was reported to induce aggressive lymphomagenesis via a p53-independent mechanism [111]. Xie et al. showed that the suppression of NONO expression modulates alternative splicing of SETMAR, resulting in lymph node metastasis progression in bladder cancer [112]. Downregulation of PTBP1 exerts anti-tubulin chemotherapeutics resistance in cancer cells by apoptotic evasion [113].

## 4. Materials and Methods

### 4.1. Acquisition of Transcriptome Dataset

RNA-Seq data samples of breast cancer patients sensitive and resistant to neoadjuvant chemotherapy were downloaded from the GEO database, GSE162187 [114] and GSE163882 [115]. A total of 89 data samples sensitive to treatment and 154 data samples resistant to the treatment were used for downstream analysis.

### 4.2. Read Mapping and Differential Gene Expression Analysis

All the samples were mapped to the human reference genome (hg38) using STAR aligner [116]; subsequently, reads mapped to each gene were calculated for all the samples using featureCounts [117]. Read count matrices were further used for differential gene expression (DGE) analysis using DESeq2 [118]. While performing DGE analysis, patients sensitive to treatment were considered as a reference. Upregulated genes were considered with *p*-values < = 0.05 and log2FoldChange > = 1, while downregulated genes were considered with *p*-values < = 0.05 and log2FoldChange < = −1.

### 4.3. Weighted Gene Co-Expression Network Analysis

Weighted gene co-expression network analysis (WGCNA) [119] detects network modules consisting of highly correlated genes, in terms of expression, that are potentially involved in similar biological processes. A total of 1446 differentially expressed genes were used for this analysis. The co-expression network was constructed using the WGCNA package [119]. The hierarchical clustering and dynamic tree cut algorithm were used to identify the co-expression modules whose gene expression was highly correlated with the clinic traits. Module-trait associations were calculated to identify the significant modules related to clinical traits. Modules that were significantly correlated with individual traits (*p*-value < 0.05) were selected, and we exported the genes of this module for further analysis.

### 4.4. Construction of PPI Network and Hub Gene Analysis

STRING v11.0 (https://string-db.org, accessed on 6 June 2022) [34] was used to construct a protein–protein Interaction (PPI) network of genes extracted from the significant WGCNA module. *Homo sapiens* was selected as an organism of interest, the minimum required interaction score was set to high confidence (0.7), and the rest of the parameters were used as default. The resultant network was visualized using Cytoscape v3.8.0 [120]. The MCC (Multiple Correlation Clustering) algorithms of the cytoHubba plug-in [121] of Cytoscape were implemented to identify the top 10% of hub genes from the network.

### 4.5. mRNA-miRNA Network Analysis

We used miRNet 2.0 [44] to construct the mRNA–miRNA network. We uploaded the hub genes to the miRNet 2.0 web server (https://www.mirnet.ca/upload/GeneUploadView.xhtml, accessed on 7 October 2022) and selected the breast cancerous tissues to extract the potential mRNA–miRNA network consisting of these hub genes.

### 4.6. Functional Enrichment Analysis of Hub Genes and miRNAs

The identified hub genes from the corresponding network were subjected to enrichment analysis. We used ShinyGo v0.76 [122] for biological processes, molecular function, KEGG, and reactome pathway enrichment. Functional enrichment of miRNAs was performed using miEAA 2.0 (https://www.ccb.uni-saarland.de/mieaa2, accessed on 7 October 2022) [45].

### 4.7. Survival Analysis of Hub Genes

Kaplan–Meier plotter analysis was performed online at https://kmplot.com/analysis, accessed on 7 October 2022 [53] and used to assess the effects of 33 hub genes on breast cancer survival. Notably, 15 hub genes were selected based on the *p*-value cutoff < 0.05.

### 4.8. Drug-hub Gene Interaction and Drug Repositioning

The Drug–Gene Interaction database (DGIdb, https://www.dgidb.org/; v4.2.0-sha1 afd9f30b, accessed on 16 July 2022) [123], Genomics and Drugs integrated Analysis (GDA, http://gda.unimore.it/, accessed on 16 July 2022) [124], drug repurposing database CLUE (https://clue.io/, accessed on 16 July 2022), and Comparative Toxicogenomics Database (CTD) (http://ctdbase.org/, accessed on 16 July 2022) [125] were used to identify FDA approved drugs for potential repurposing.

## 5. Conclusions

In this study, we identified the hub genes associated with developing resistance to NAC in breast cancer and potential repurposing drugs for overcoming resistance. Our study provides detailed molecular insight into NAC resistance in breast cancer. However, the main limitation of the study is that we did not experimentally validate any of the selected hub genes, as our findings are mainly based on the public dataset. Furthermore, the drug repurposing described here is based on preliminary studies and requires further experimental verification and proper clinical trials. Therefore, the findings of the present study encourage the further design of experimental research to understand the molecular mechanisms behind the NAC resistance in breast cancer, which could facilitate the development of precision medicine in the near future.

## Data Availability

Not applicable.

## Supplementary Materials

The following supporting information can be downloaded at: https://www.mdpi.com/article/10.3390/ijms232012628/s1.
