# Supplementary material for: Identifying Hub Genes Associated with Neoadjuvant Chemotherapy Resistance in Breast Cancer and Potential Drug Repurposing for the Development of Precision Medicine
Source: Int J Mol Sci. 2022 Oct 20;23(20):12628. doi: 10.3390/ijms232012628 (PMC9603969; doi:10.3390/ijms232012628)
Supplement: Supplementary file 1 [file ijms-23-12628-s001.zip › Supplementary_Information_legends.pdf]

Supplementary Information:

Figure S1: *Kaplan-Meier survival curves of 15 hub genes (p-Value cutoff <0.1) differentially expressed in NAC resistant breast cancer patients. Overall survival (OS) by low (black line) and high (red line) (A) ITGB1, (B) PSMB5, (C) SEC61A1, (D) RPSA, (E) PSME3, (F) SNRNP70, (G) SRSF3 expression.*

Table S1: *Experimental evidence of hub genes and miRNAs interactions.*

Table S2: *List of differentially expressed hub genes and their FDA approved drugs.*

Table S3: *List of manually validated FDA approved drugs from Comparative Toxicogenomics Database (CTD) for drug repurposing to target hub genes.*
