# Supplementary figures and images for: Identifying Hub Genes Associated with Neoadjuvant Chemotherapy Resistance in Breast Cancer and Potential Drug Repurposing for the Development of Precision Medicine
Source: Int J Mol Sci. 2022 Oct 20;23(20):12628. doi: 10.3390/ijms232012628 (PMC9603969; doi:10.3390/ijms232012628)

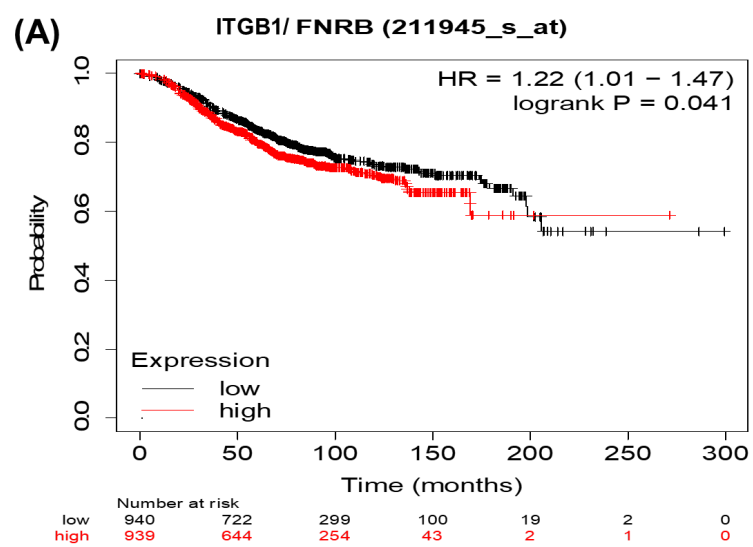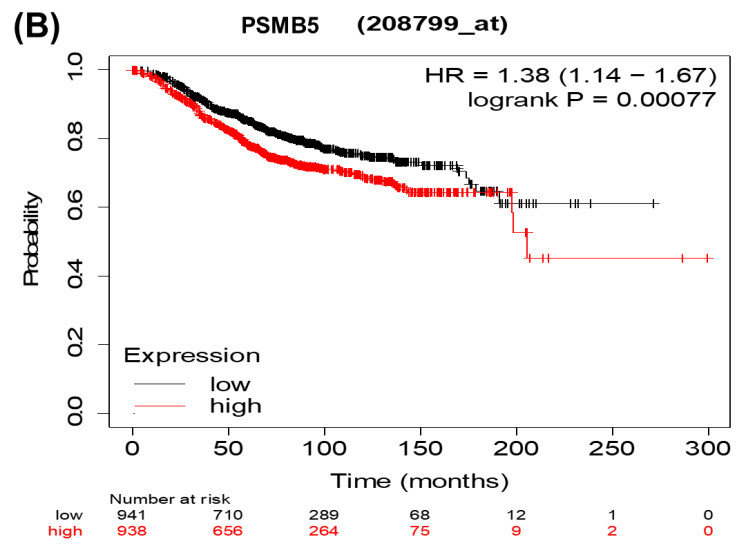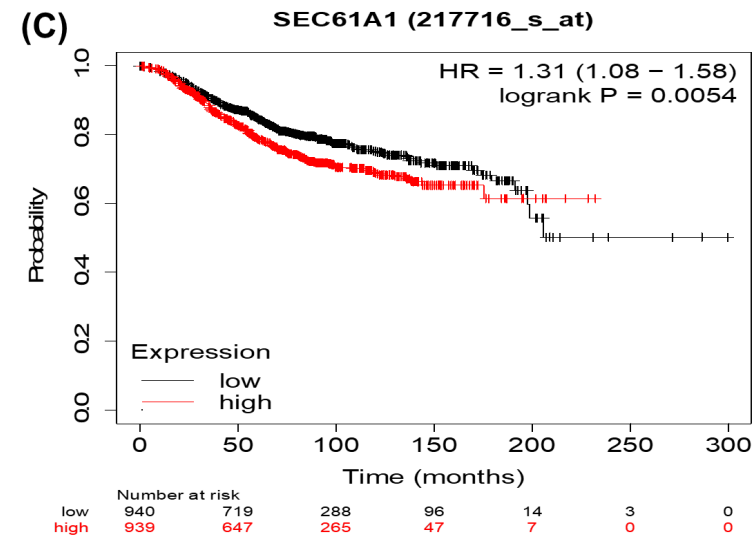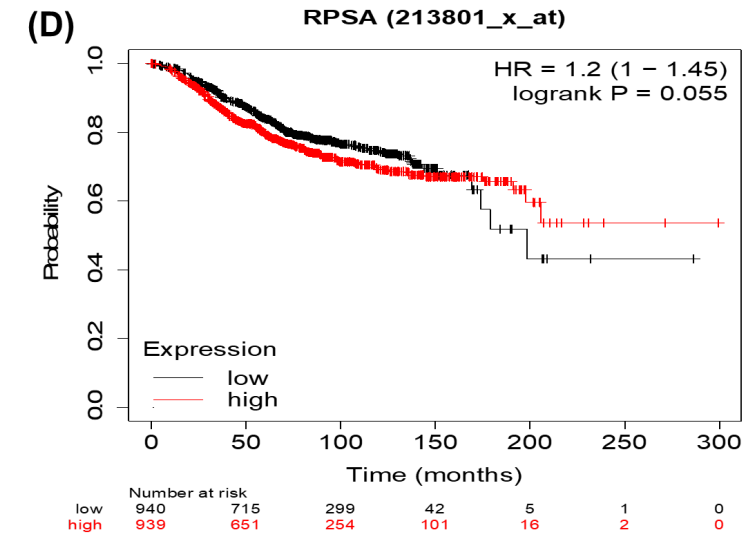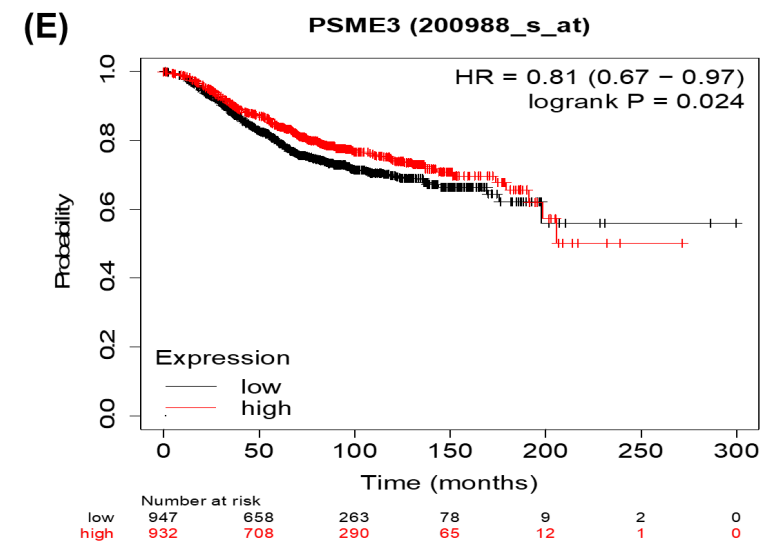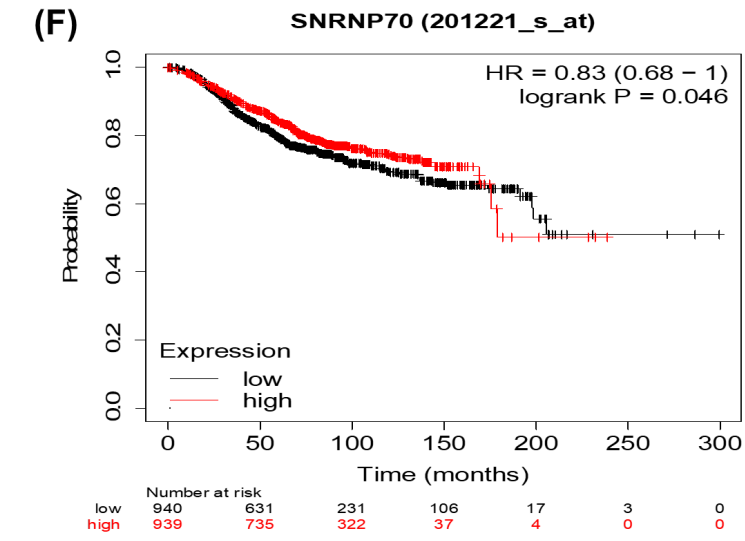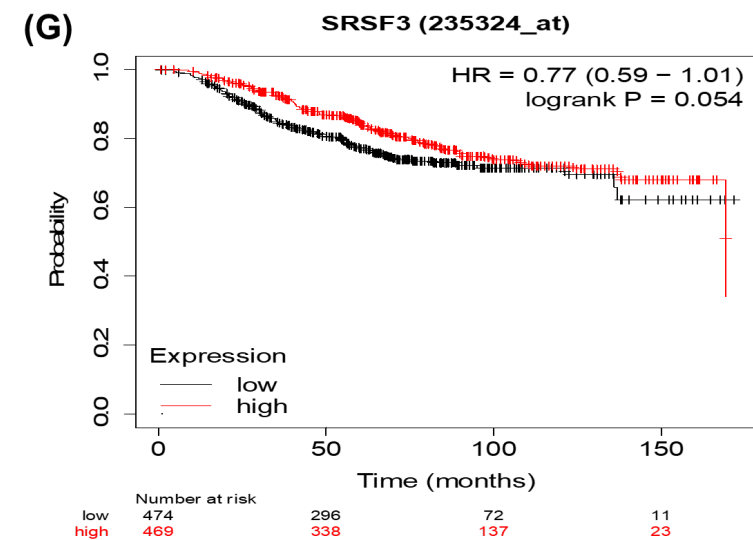

Supplement: Supplementary file 1 [file ijms-23-12628-s001.zip › Figure S1.pdf]
